# Opportunity Cost of Environmental Regulation in China’s Industrial Sector

**DOI:** 10.3390/ijerph18168579

**Published:** 2021-08-13

**Authors:** Ye Wang, Yunguo Lu, Lin Zhang

**Affiliations:** 1 School of Russian Studies, Dalian University of Foreign Languages, Dalian 116044, China; wangye@dlufl.edu.cn; 2 School of Energy and Environment, City University of Hong Kong, Hong Kong, China; 3 Department of Public Policy, College of Liberal Arts and Social Sciences, City University of Hong Kong, Hong Kong, China

**Keywords:** environmental regulation, opportunity cost, technical change, input change, mediation effect

## Abstract

In this paper, we employ a directional distance function to estimate the opportunity cost arising from environmental regulations in China’s industrial sector. The change of opportunity cost is decomposed mathematically into two components including technical change and input change. Our results show that the opportunity cost attributed to environmental regulation is nil in some regions. The change of opportunity cost is marginal at the national level, as the positive effect of technical change is canceled out by the negative impact of input change on opportunity cost. Built on our mathematical decomposition, we further estimate the effects of environmental regulations on opportunity cost using a mediation model. It shows that environmental regulation has a significantly positive direct effect and a significantly negative indirect effect through foreign direct investment on opportunity cost. Our findings suggest, firstly, that inward FDI in China’s industrial sector represents relatively dirty production technology; and, secondly, industrial production has transited towards a less carbon-intensive input mix. This paper, therefore, provides new insights for the recent dynamics of carbon abatement performance of China’s industrial sector with policy implications.

## 1. Introduction

The industrial sector is China’s biggest energy consumer and largest carbon emitter. It accounted for over 65% of total energy consumption and over 70% of total carbon emissions. Therefore, a series of measures and reduction targets have been adopted to improve energy efficiency and reduce carbon emissions in Chinese industry. According to the 13th Five-Year Plan for National Economic and Social Development, the carbon dioxide emissions per unit of GDP should be reduced by 18% and energy consumption per unit of GDP should drop by 15% compared with the corresponding levels in 2015. At the same time, the 13th Five-Year Plan also highlights the importance of effectively controlling carbon emissions from key industries such as electricity, steel, building materials and chemicals, and promoting low-carbon development in these key sectors. In addition, in 2015, China signed the Paris Agreement on climate change and announced the new CO_2_ reduction target, that is, by 2030, to achieve a 60–65% reduction in carbon intensity compared with the level of 2005. Such stringent regulations will change the costs of carbon abatement. This paper attempts to identify the extent to which such environmental regulations affect the cost of carbon abatement as well as the driving factors underlying the changes.

In this study, we estimate the opportunity cost arising from environmental regulations for the industrial sector in China based on directional output distance function. We further decompose the change of opportunity cost into two contributing factors, i.e., input change and technical change. To further explore the relationship among environmental regulations, technical change, input change and opportunity cost, we propose a mediation model to analyze the direct and indirect effects of environmental regulations on opportunity cost.

Our study is related to two strings of literature. First, this paper complements to the literature on analyses of abatement costs and the drivers of abatement costs change. Abatement costs associated with pollution abatement activities or environmental regulations in literature can be estimated either by measuring a cost function [1,2,3] or deriving a shadow price based on Shephard output distance function [4,5,6,7]. However, the former depends on reliable cost information which is usually confidential and unavailable; the latter one is subject to the issue of projecting the observations to the production frontier by increasing good and bad outputs proportionally. A third option is to apply the directional output distance function approach proposed by Chung et al. [8], which has been adopted for estimating marginal abatement costs of pollution emissions in many countries and industries, including Färe et al. [9], Lee and Zhou [10] for the U.S., Murty et al. [11] for India, Park and Lim [12] for Korea, Matsushita and Yamane [13] for Japan, Wei et al. [14], Ma et al. [15,16] for China, etc. We apply this approach to estimate the opportunity cost arising from environmental regulations as it is much more flexible and allows us to estimate the distance to the production frontier by increasing good outputs and reducing bad outputs simultaneously. We then decompose the change of OC into two driving factors. The results show that opportunity cost attributed to environmental regulation is zero for the industrial sector in some regions during the sample period. Moreover, there is no significant change on the opportunity cost at the national level, which is due to the fact that the positive effect of technical change on opportunity cost is cancelled out by the negative effect of input change during the entire period.

In addition, this paper contributes to the understanding of the economic impacts of environmental regulation. Some previous studies have estimated the cost of pollution abatement activities or environmental regulation. Boyd and McClelland [17] find that environmental constraints which prohibit paper plants from freely disposing of pollutants can cost firms between 4.3% and 10.1% in good output value using hyperbolic analysis. Pasurka [18] estimated the costs associated with environmental regulation and discussed the effect of technical change on abatement costs using an activity analysis framework. Picazo-Tadeo et al. [19] estimated the opportunity costs of environmental regulations for Spanish producers of ceramic pavements based on directional distance function. Similarly, Färe et al. [20,21] employed environmental production function to analyze the pollution abatement costs of coal-fired power plants in the USA from the perspective of opportunity cost. We further explored the direct effect and the indirect effect of environmental regulations on opportunity cost through technical change based on a mediation model, where technical change is the mediator. The results show that environmental regulations have a significantly positive direct effect and a significantly negative indirect effect on opportunity cost through the technology spillover of foreign direct investment. These provide a novel perspective for us to understand the impact of environmental regulations on opportunity cost in China’s industrial sector. Thus, this study can be a good supplement to previous studies.

The remainder of the paper is organized as follows. Section 2 presents the methodology, followed by the description of the data and variables. Section 4 reports and discusses the results. The last section concludes the paper.

## 2. Methodology

### 2.1. Defining Opportunity Cost of Environmental Regulation

Assume the industrial sector in region *k* (*k* = 1, …, *K*) uses a vector of *N* inputs $x=(x_{1},\ldots ,x_{N}$)$\in R_{+}^{N}$ to produce a vector of good outputs $y=(y_{1},\ldots ,y_{M})\in R_{+}^{M}$ and a vector of bad outputs $b=\left(b_{1},\ldots ,b_{J}\right)\in R_{+}^{J}$, where *M* and *J* are the number of good and bad outputs, respectively. This can be represented by the output set ***P*** which can be defined as follows:

(1) P(x)={(y,b):x can produce(y,b)}

The output set ***P*** indicates that the goods **(*y*)** and bad outputs **(*b*)** can be jointly produced using the input vector ***x***. A series of assumptions are required to satisfy the joint production model (Färe et al., 2005). In addition to the traditional convex and compact assumptions, output set ***P*** should also satisfy free or strong disposability in inputs, i.e., if $x^{*}>x,\;\mathrm{then}\;P\left(x\right)\subseteq P\left(x^{*}\right)$. This assumption suggests that inputs are freely disposable. The assumption of null-jointness is also required, that is, if (y,b)∈P(x) and b=0, then y=0, which indicates that it is impossible to produce any good output without any bad output. Additionally, the good outputs and bad outputs are required to satisfy joint weak disposability assumption, i.e., if (y,b)∈P(x),and 0≤θ≤1,then (θy,θb)∈P(x), which indicates that it is feasible to reduce good and bad outputs proportionally. Finally, the good outputs are freely or strongly disposable, i.e., if (y,b)∈P(x) and $y^{\prime }\leq y,\;\mathrm{then}\;\left(y^{\prime },b\right)\in P\left(x\right)$. This suggests that the good outputs can be freely disposable without reducing the bad outputs.

To estimate the opportunity cost of environmental regulation for the industry in China, we resort to the directional output distance function, which can be represented as follows:

(2) $${\vec{D}}_{0}\left(x,y,b;g_{y^{,}}-g_{b}\right)=\mathrm{Max}\{\beta :\left(y+\beta g_{y^{,}}b-\beta g_{b}\right)\epsilon P\left(x\right)\}$$

where the mapping rule of the observations is given by direction vector $g=\left(g_{y^{,}}-g_{b}\right)$, i.e., the directions along which good outputs are expanded and bad outputs are contracted simultaneously to reach the frontier.

We then introduce the regulated technology and unregulated technology. According to Picazo-Tadeo et al. [19] and Färe et al. [20,21], the opportunity cost of pollution abatement activities is measured by the difference of good outputs with unregulated and regulated technologies, respectively. In this paper, CO_2_ emissions is the bad output, and the industry can freely dispose of CO_2_ emissions if completely unregulated. Thus, with the unregulated technology, both the gross industrial product value (good output) and CO_2_ emissions (bad output) are freely disposable for the industry. However, with the regulated technology, CO_2_ emissions are weakly disposable. The difference of the good outputs with the regulated and unregulated technologies is defined as the opportunity costs of carbon abatement activities or regulations.

Following Picazo-Tadeo et al. [19] and Färe et al. [20], we estimate the unregulated directional distance function (i.e., industrial sector without regulations on CO_2_ emissions) for the industry in region *k*′ as:

(3) $${\vec{D}}^{u}\left(x^{k}{}^{\prime },y^{k}{}^{\prime },b^{k}{}^{\prime };g_{y^{,}}-g_{b}\right)=\mathrm{Max}\;\beta$$

$$\;s.t.\;\sum_{k=1}^{K}z^{k}y_{m}^{k}\geq y_{m}^{k^{\prime }}+\beta y_{m}^{k^{\prime }},\;m=1,\ldots ,M$$

$$\;\sum_{k=1}^{K}z^{k}b_{j}^{k}\geq b_{j}^{k^{\prime }}-\beta b_{j}^{k^{\prime }},\;j=1,\ldots ,J$$

$$\;\sum_{k=1}^{K}z^{k}x_{n}^{k}\leq x_{n}^{k^{\prime }},\;n=1,\ldots ,N$$

$$\sum_{k=1}^{K}z^{k}=1,\;$$

$$\;z^{k}\geq 0,\;k=1,\ldots ,K$$

where *z* = ($z^{1},\ldots ,z^{k})$ is a *K* × 1 vector of intensity variables, which indicates the intensity assigned to each observation for constructing the production set. The second set of constraints on the bad outputs imposes free disposability assumption, that is, the production of bad output is unregulated, suggesting that the industry can freely dispose of bad outputs without any cost. The fourth constraint represents that variable returns to scale (VRS) is imposed. If the parameter β equals zero, it suggests that the observation is located on the unregulated production possibilities frontier, that is, the observation is technically efficient. This study chooses the directional vector as *g* = (*y*, −*b*).

The regulated directional distance function can be represented as:

(4) $${\vec{D}}^{r}\left(x^{k}{}^{\prime },y^{k}{}^{\prime },b^{k}{}^{\prime };g_{y^{,}}-g_{b}\right)=\mathrm{Max}\;\beta^{r}$$

$$\;s.t.\;\sum_{k=1}^{K}z^{k}y_{m}^{k}\geq y_{m}^{k^{\prime }}+\beta y_{m}^{k^{\prime }},\;m=1,\ldots ,M$$

$$\;\sum_{k=1}^{K}z^{k}b_{j}^{k}=b_{j}^{k^{\prime }}-\beta b_{j}^{k^{\prime }},\;j=1,\ldots ,J$$

$$\;\sum_{k=1}^{K}z^{k}x_{n}^{k}\leq x_{n}^{k^{\prime }},\;n=1,\ldots ,N$$

$$\sum_{k=1}^{K}z^{k}=1,\;$$

$$\;z^{k}\geq 0,\;k=1,\ldots ,K$$

The difference between Equations (3) and (4) is that Equation (4) imposes a set of equality constraints for the bad outputs while Equation (3) does not. The equality constraints represent that the production of bad outputs is regulated, showing that the bad outputs are reduced at the cost of a smaller production of good outputs.

Picazo-Tadeo et al. [19] and Färe et al. [20] defined the opportunity cost as the difference of maximum good outputs with unregulated and regulated technologies, i.e., ${OC}^{k}{}^{\prime }=y_{u}^{k}{}^{\prime }-y_{r}^{k}{}^{\prime },$ which is the loss of good outputs associated with pollution abatement activities or environmental regulations. Following their definitions, we define the opportunity cost as the ratio of maximum good outputs under unregulated and regulated technologies, which provides a frontier reference for possible compliance cost of carbon emissions mitigation regulations. This definition makes it possible to further investigate the factors affecting the change of the opportunity costs. Specifically, the opportunity cost for the industry in region $k^{\prime }$ can be written as:

(5) $${OC}^{k}{}^{\prime }=\frac{y_{u}^{k}{}^{\prime }}{y_{r}^{k}{}^{\prime }}=\frac{y^{k}{}^{\prime }+{\vec{D}}^{u}\left(x^{k}{}^{\prime },y^{k}{}^{\prime },b^{k}{}^{\prime };g_{y^{,}}-g_{b}\right)y^{k}{}^{\prime }}{y^{k}{}^{\prime }+{\vec{D}}^{r}\left(x^{k}{}^{\prime },y^{k}{}^{\prime },b^{k}{}^{\prime };g_{y^{,}}-g_{b}\right)y^{k}{}^{\prime }}$$

where $y^{k}{}^{\prime }$ is the actual good output of the industrial sector in region *k*′, and the subscript *u* and *r* represent unregulated and regulated technology, respectively.

The *OC* measures the opportunity cost of carbon abatement activities, that is, the reduced good output (gross industrial product value) when carbon emissions are not freely disposable. If the value of *OC* equals one, it means that there is no reduced good output due to carbon abatement activities or regulations. When the value of *OC* is greater than one, it implies that abatement activities or regulations have reduced industrial output.

**Figure 1:**
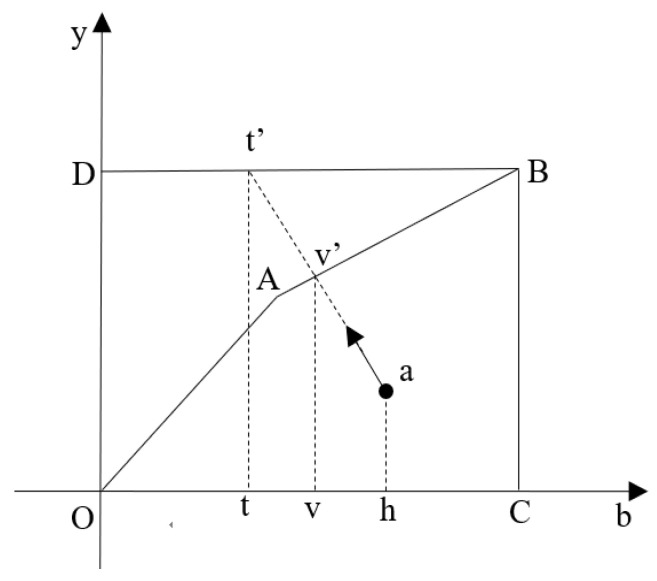
Graphical illustration of opportunity cost.

Figure 1 explains how opportunity cost is defined graphically. In the figure, the unregulated production set *ODBC* and the regulated production set *OABC* represent the feasible combinations of good and bad outputs that can be produced for the given inputs and technology. A production combination *a* will be projected to point *t*′ when the bad output is unregulated, but it will be projected to point *v*′ when the bad output is regulated. According to the definition, opportunity cost is defined as *tt*′/*vv*′. If opportunity cost were defined to be *tt*′/*ah*, it includes the reduced good output due to technical inefficiency, which is not the purpose of this paper.

### 2.2. Decomposing the Change of Opportunity Cost

We can further analyze the change in opportunity cost between period *t* and *t* + 1 based on the amount of foregone industrial output due to the regulation of CO_2_ emissions. Formally, the change of opportunity cost between period *t* and *t* + 1 is defined as:

(6) $$\Delta OC_{t}^{t+1}=\frac{OC^{t+1}}{OC^{t}}=\frac{\left(y_{u}^{t+1}/y_{r}^{t+1}\right)}{\left(y_{u}^{t}/y_{r}^{t}\right)}=\frac{\left(y_{u}^{t+1}/y_{u}^{t}\right)}{\left(y_{r}^{t+1}/y_{r}^{t}\right)}$$

$\Delta OC_{t}^{t+1}$ implies the change in industrial output forgone due to the carbon abatement activities or regulations on carbon emissions from period *t* to period *t* + 1. If the value of $\Delta OC_{t}^{t+1}$ equals one, opportunity cost remains the same from period *t* to period *t* + 1. A value of more (less) than one indicates that opportunity cost has increased (decreased) from period *t* to period *t* + 1.

**Figure 2:**
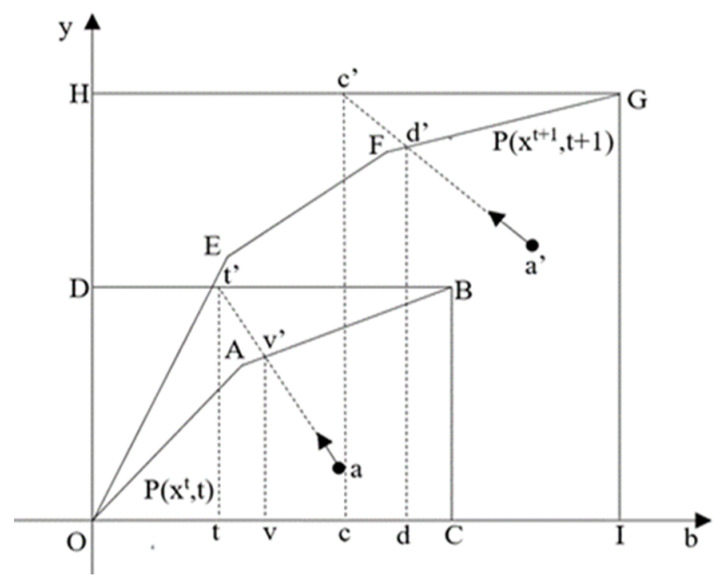
Change of opportunity cost.

Figure 2 shows how the change of opportunity cost is defined graphically. In the figure, the regulated production frontiers in period *t* and *t* + 1 are *OABC* and *OEFGI* respectively, while the unregulated production frontiers in period *t* and *t* + 1 are *ODBC* and *OHGI* respectively. Point *a* is the observation in period *t* and point *a*′ is the output combination in period *t* + 1. *tt*′/*vv*′ is the opportunity cost in period *t* while *cc*′/*dd*′ is the opportunity cost in period *t* + 1. Thus, the change of opportunity cost is represented as: $\Delta OC_{t}^{t+1}=\frac{cc^{\prime }/dd^{\prime }}{tt^{\prime }/vv^{\prime }}$. We can observe that whether the opportunity cost (which is represented as the distance between the unregulated and regulated production frontiers) increases or decreases depends on the relative shifts of the unregulated and regulated frontiers.

The advances in production technology can help the unregulated output frontier expand outward from period *t* to *t* + 1. Facing regulations on CO_2_ emissions, the industrial sector seeks to develop the regulated technology which expand industrial output and reduce carbon emissions simultaneously such that the regulated output frontier will also expand outward. On the other hand, different combinations of inputs will lead to different industrial production and carbon emissions located at the frontiers. These will bring two effects on opportunity cost: (1) a change in opportunity cost due to technical change and (2) a change in opportunity cost due to input change. Equation (6) can be further decomposed to capture these two separate effects. To avoid choosing an arbitrary base period, we use the geometric mean of period *t* and period *t* + 1 as the reference technology:

(7) $$\begin{array}{cc} \Delta OC_{t}^{t+1} & =\frac{y_{u}^{t+1}\left(X^{t+1}\right)/y_{u}^{t}\left(X^{t}\right)}{y_{r}^{t+1}\left(X^{t+1}\right)/y_{r}^{t}\left(X^{t}\right)}\\ & =\left([\frac{y_{u}^{t+1}\left(X^{t+1}\right)/y_{u}^{t}\left(X^{t+1}\right)}{y_{r}^{t+1}\left(X^{t+1}\right)/y_{r}^{t}\left(X^{t+1}\right)}{]}^{\frac{1}{2}}\times [\frac{y_{u}^{t+1}\left(X^{t}\right)/y_{u}^{t}\left(X^{t}\right)}{y_{r}^{t+1}\left(X^{t}\right)/y_{r}^{t}\left(X^{t}\right)}{]}^{\frac{1}{2}}\right)\\ & \times \left([\frac{y_{u}^{t}\left(X^{t+1}\right)/y_{u}^{t}\left(X^{t}\right)}{y_{r}^{t}\left(X^{t+1}\right)/y_{r}^{t}\left(X^{t}\right)}{]}^{\frac{1}{2}}\times [\frac{y_{u}^{t+1}\left(X^{t+1}\right)/y_{u}^{t+1}\left(X^{t}\right)}{y_{r}^{t+1}\left(X^{t+1}\right)/y_{r}^{t+1}\left(X^{t}\right)}{]}^{\frac{1}{2}}\right)\\ & =\frac{TC_{u}}{TC_{r}}\times \frac{IC_{u}}{IC_{r}}\\ & =\Delta OC_{TC}\times \Delta OC_{IC} \end{array}$$

where $X^{t}$ and $X^{t+1}$ represent the inputs at period *t* and *t* + 1 respectively. In Equation (7), $y_{u}^{t+1}\left(X^{t+1}\right)$ is the maximum unregulated good output with both the inputs and technology in period *t* + 1. Similarly, $y_{u}^{t}\left(X^{t}\right)$ is the maximum unregulated good output with both the inputs and technology in period *t*. By contrast, $y_{u}^{t}\left(X^{t+1}\right)$ is the maximum unregulated good output with the inputs in period *t* + 1 while the technology in period *t*, and $y_{u}^{t+1}\left(X^{t}\right)$ is the maximum unregulated good output with the inputs in period *t* while the technology in period *t* + 1. Likewise, we can obtain the corresponding maximum regulated good output by substituting the subscript *u* by *r*.

The term in first parentheses (i.e., $\Delta OC_{TC}=\frac{TC_{u}}{TC_{r}}=[\frac{y_{u}^{t+1}\left(X^{t+1}\right)/y_{u}^{t}\left(X^{t+1}\right)}{y_{r}^{t+1}\left(X^{t+1}\right)/y_{r}^{t}\left(X^{t+1}\right)}{]}^{\frac{1}{2}}\times [\frac{y_{u}^{t+1}\left(X^{t}\right)/y_{u}^{t}\left(X^{t}\right)}{y_{r}^{t+1}\left(X^{t}\right)/y_{r}^{t}\left(X^{t}\right)}{]}^{\frac{1}{2}}$) indicates that the change in opportunity cost is related to the technical change. The term in the second parentheses (i.e., $\Delta OC_{IC}=\frac{IC_{u}}{IC_{r}}=[\frac{y_{u}^{t}\left(X^{t+1}\right)/y_{u}^{t}\left(X^{t}\right)}{y_{r}^{t}\left(X^{t+1}\right)/y_{r}^{t}\left(X^{t}\right)}{]}^{\frac{1}{2}}\times [\frac{y_{u}^{t+1}\left(X^{t+1}\right)/y_{u}^{t+1}\left(X^{t}\right)}{y_{r}^{t+1}\left(X^{t+1}\right)/y_{r}^{t+1}\left(X^{t}\right)}{]}^{\frac{1}{2}}$) suggests that the change in opportunity cost is related to the input change. Specifically, $\frac{TC_{u}}{TC_{r}}$ represents the change of good output because of technical change in the unregulated technology relative to the change of good output due to technical change in the regulated technology. Similarly, $\frac{IC_{u}}{IC_{r}}$ is the change of good output with unregulated technology because of input changes relative to the change of good output with regulated technology due to input change. Therefore, the term $\Delta OC_{t}^{t+1}$ can be decomposed into two parts, that is, $\Delta OC_{TC}$ and $\Delta OC_{IC}$. If the value of $\Delta OC_{TC}$ is lower (higher) than unity, it means that opportunity cost has decreased (increased) from period *t* to *t* + 1 due to technical change. Similarly, if the value of $\Delta OC_{IC}$ is lower (higher) than unity, it means that opportunity cost has decreased (increased) from period *t* to *t* + 1 due to input change.

**Figure 3:**
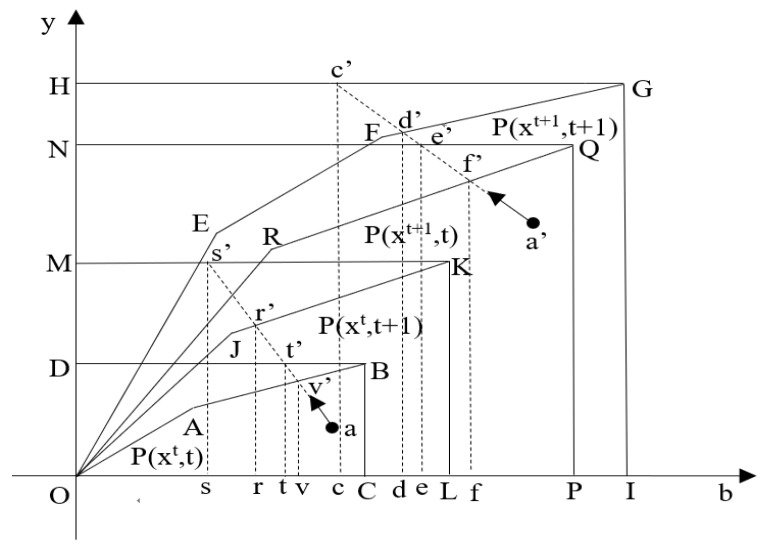
Decomposition of the change of opportunity cost.

Figure 3 illustrates the decomposition of Δ*OC* graphically. The regulated frontier (ORQP) and the unregulated frontier (ONQP) are the combinations of good and bad outputs that can be produced using the inputs in period *t* + 1 with the technology in period *t*. The unregulated frontier (OJKL) and regulated frontier (OMKL) are the combinations of good and bad outputs that can be produced using the inputs levels in period *t* with the technology in period *t* + 1.

According to Equation (7), the change of opportunity cost from period *t* to *t* + 1 can be rewritten as:

(8) $$\begin{array}{cc} \Delta OC_{t}^{t+1} & =\frac{cc^{\prime }/tt^{\prime }}{dd^{\prime }/vv^{\prime }}\\ & =\left([\frac{cc^{\prime }/ee^{\prime }}{dd^{\prime }/ff^{\prime }}{]}^{\frac{1}{2}}\times [\frac{ss^{\prime }/tt^{\prime }}{rr^{\prime }/vv^{\prime }}{]}^{\frac{1}{2}}\right)\times \left([\frac{ee^{\prime }/tt^{\prime }}{ff^{\prime }/vv^{\prime }}{]}^{\frac{1}{2}}\times [\frac{cc^{\prime }/ss^{\prime }}{dd^{\prime }/rr^{\prime }}{]}^{\frac{1}{2}}\right)\\ & =\frac{TC_{u}}{TC_{r}}\times \frac{IC_{u}}{IC_{r}}\\ & =\Delta OC_{TC}\times \Delta OC_{IC} \end{array}$$

The decomposition analysis defined in Equation (7) requires a set of the maximum good output *y*, which can be obtained by using the directional distance function and we show the detailed calculations in Appendix A.

### 2.3. Mediation Model

In the previous section, we have decomposed the change in opportunity cost arising from environmental regulations into technical change and input change. Generally, when facing environmental regulation, firms or industries will not reduce the input, which could reduce the final output and thus can influence the profits. Instead, firms or industries always resort to advanced production technology or abatement technology to reduce the emissions emitted in the production process. A large number of studies have explored the response of technology to environmental regulations theoretically and empirically, examples of which include Goulder and Mathai [22], Buonanno et al. [23], Fischer and Newell [24], Grimaud and Rouge [25], Carraro et al. [26], and Acemoglu et al. [27].

**Figure 4:**
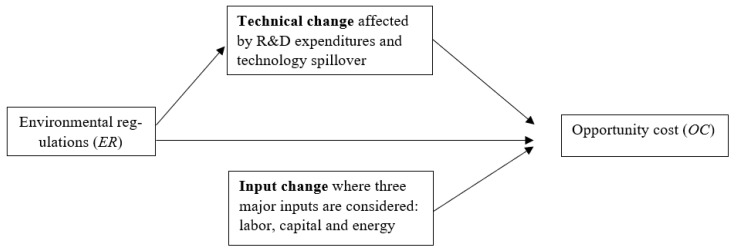
Mediation model of environmental regulations and opportunity cost.

In this study, we develop a mediation model to further analyze the effects of environmental regulations on opportunity cost. According to Figure 4, there are two pathways by which environmental regulations can affect opportunity cost. One pathway leads from environmental regulations to opportunity cost without passing through technical change, which is the *direct* effect. Another pathway from environmental regulations to opportunity cost through technical change is the *indirect* effect, where technical change is the mediator. In addition, in this paper, we assume the separability condition holds, i.e., the explanatory variables in the second stage affect only the distance from the frontier (distribution of efficiency) but not the shape of the frontier (production possibilities), as done in Liu et al. [28]. Simar and Wilson [29] pointed out this may not be realistic. The recent study by Daraio et al. [30] developed a test of the ‘separability’ condition that is necessary for second-stage regressions of efficiency estimates on environmental variables. A general fully non-parametric way to treat appropriately the presence of environmental factors in a production process is the probabilistic approach which is well documented in the studies by Bădin et al. [31,32,33,34].

Previous studies have shown that technical change is highly related to the R&D expenditures and foreign direct investment (FDI), i.e., R&D expenditures can promote the innovation and thus can help the industrial technical change and FDI has a technology spillover effect on the industry in host country. For example, Perelman [35] used the R&D variable and an indicator of international trade to reflect the technical change of productivity growth for OECD industrial sectors. Under the pressure of regulations, higher R&D expenditures will be used to develop the clean technology, i.e., regulated technology in this paper, thus can help to decrease OC. In literature, FDI may bring advanced clean/green technology to the host country, otherwise it may represent the unregulated technology, i.e., heavy-polluting technology according to the pollution heaven hypothesis, which can lead to the increase of OC. Therefore, we employ these two variables to represent the technical change of China’s industrial sector. Furthermore, the estimation of the mediation model can be represented as follows:

(9) $$\ln FDI_{kt}=\alpha_{FDI}+\alpha_{1}\ln ER_{kt}+\beta_{2}\ln Labor_{kt}+\beta_{3}\ln Energy_{kt}+\beta_{4}\ln Capital_{kt}+\gamma_{k}+\mu_{t}+\varepsilon_{kt}$$

(10) $$\ln RD_{kt}=\alpha_{RD}+\alpha_{2}\ln ER_{kt}+\beta_{2}\ln Labor_{kt}+\beta_{3}\ln Energy_{kt}+\beta_{4}\ln Capital_{kt}+\gamma_{k}+\mu_{t}+\varepsilon_{kt}$$

(11) $$\begin{array}{c} \ln OC_{kt}=\beta_{0}+\beta_{1}\ln\;ER_{kt}+\beta_{2}\ln Labor_{kt}+\beta_{3}\ln Energy_{kt}+\beta_{4}\ln Capital_{kt}+\beta_{5}\ln FDI_{kt}\\ +\beta_{6}\ln R\&D_{kt}+\gamma_{k}+\mu_{t}+\varepsilon_{kt} \end{array}$$

where subscripts *k* and *t* represent the industry is in province *k* in year *t*. $\alpha_{1}$ and $\alpha_{2}$ estimate the effect of environmental regulation on FDI and R&D of the industry. $\beta_{1}$ measures the direct effect of environmental regulation on opportunity cost. $\beta_{2}$~$\beta_{6}$ measures the effects of energy, labor, capital, FDI, R&D on opportunity cost respectively. $\alpha_{1}\beta_{5}$ and $\alpha_{2}\beta_{6}$ represent the indirect effect of the environmental regulation on opportunity cost through FDI and R&D. Adding these specific indirect effects will yield the total indirect effects of the environmental regulation on opportunity cost through all mediators (FDI and R&D). Input variables are treated as control variables in Equations (9) and (10), Equations (9)–(11) control the provincial fixed effects ($\gamma_{k}$) and time fixed effects ($\mu_{t}$), respectively. Obviously, the coefficients for the regression represent the percent change of dependent variables when the independent variables differ by 1%.

## 3. Data and Variables

Provincial-level industrial data are employed in this study covering the period from 2003 to 2015 in China (Tibet, Taiwan, Hong Kong and Macau are excluded due to the missing data). The three inputs for the industry are described as follows: (1) labor: measured by the annual industrial average number of employees, (2) capital: measured by the net value of fixed assets, (3) energy: measured by the total standard coal consumption of the industrial sector. The good output y is represented as the real regional gross industrial product value at a constant price in 2003. Based on the CO_2_ emission coefficients published by IPCC [36] and the National Coordination Committee Office on Climate Change and the Energy Research Institute under the National Development and Reform Commission [37], the bad output (i.e., CO_2_) is estimated using total consumption of standard coal consumption and the corresponding CO_2_ emissions factors (Du et al. [38]).

(12) $$Carbon_{it}=E_{it}\times EF$$

where *i* and *t* are the indices of province and year, respectively; $E_{it}$ is the consumption of standard coal by the industrial sector in province *i* in the year *t*; EF represents the CO_2_ emissions factor of standard coal.

According to Fosfuri et al. [39], Multinational companies can transfer technology to local firms through training employees in host countries, and then realize technology spillovers through employee flows. Thus, we use the ratio of employees in Hong Kong–Macao–Taiwan owned and foreign-owned industrial companies to the total employees in the industry to represent the technological spillovers of foreign direct investment (FDI). R&D (research and development) intensity is measured by the ratio of R&D expenditures to industrial income. Following McConnell and Schwab [40] and Cole et al. [41], we use the ratio of annual industrial waste gas treatment facilities operating cost to industrial added value to represent the environmental regulation (ER) variable.

The data for this study are collected from the China Statistical Yearbook, China Industrial Statistical Yearbook, China Energy Statistical Yearbook, China Environmental Statistical Yearbook and China Statistical Yearbook on Science and Technology. Table 1 presents the summary statistics of inputs, outputs, technical variables and environmental regulation variable in the industrial sector from 2003–2015.

## 4. Results and Discussion

### 4.1. Estimated Opportunity Cost and Its Changes

**Figure 5:**
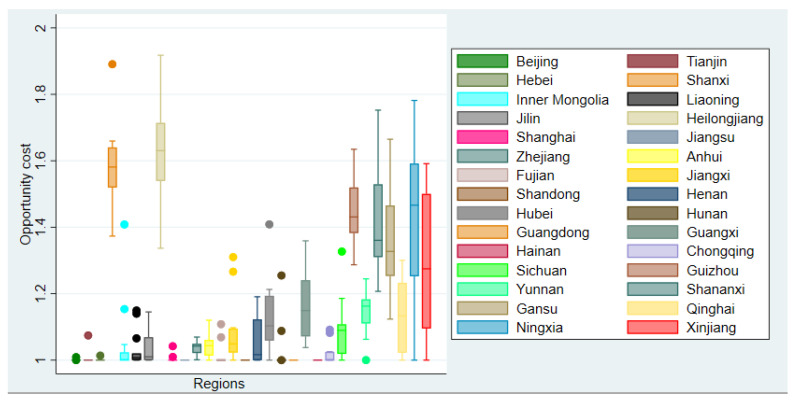
Box plot of opportunity cost from 2003 to 2015 by regions. Note: The horizontal axis represents the industrial sectors in 30 provinces of China and the vertical axis represents the opportunity cost due to environmental regulations during the sample period. The lower and upper hinge of the box present the 25th and 75th percentiles of opportunity cost, the middle line of the boxes represents the median value, and the dots represent outliers.

Figure 5 presents the boxplot of opportunity cost due to regulations of industrial sectors during the sample period by regions. Table A1 in Appendix A reports the summary statistics of estimated opportunity cost of industrial sectors in different provinces during the sample period.

We can see that the values of estimated OC are around unity for the industrial sectors in Beijing, Tianjin, Hebei, Shanghai, Jiangsu, Fujian, Shandong, Guangdong and Hainan during the sample period, which implies that there is almost no opportunity cost arising from environmental regulations, i.e., no industrial output losses due to regulations. One possible explanation is that the industrial sectors in these relatively developed regions face stronger environmental regulations which force them to develop the regulated technology in terms of less carbon emissions per unit of output, thus the maximum regulated industrial output is the same as the maximum unregulated industrial output.

In contrast to the case in these developed areas, opportunity costs in the relatively less-developed regions are much higher than unity with larger variation including Shanxi, Heilongjiang, Guizhou, Shaanxi, Gansu, Qinghai, Ningxia, Xinjiang. This suggests that industries that employ relatively backward production technology to produce products with massive amounts of carbon emissions in these regions, cannot realize the same level of maximum unregulated industrial output when they face strict regulations. Therefore, the decline in industrial output due to regulations is much higher compared with the industry in the developed areas.

Table 2 reports the geometric means of $\Delta OC,\;\Delta OC_{TC}\;\mathrm{and}\;\Delta OC_{IC}$ of the industry in provinces from 2003 to 2015. The results show that on average, the opportunity costs of Beijing, Jiangsu, Shandong, Guangdong, and Hainan remained unchanged during the period while we can observe a sustained decline of opportunity cost in some regions like Tianjin, Inner Mongolia, Hubei, etc., and a sustained increase in some provinces like Shanxi, Heilongjiang, Qinghai, etc.

Technical change is associated with increasing opportunity cost during the whole period, and the size of annual increase in opportunity cost varies across the regions. From Table A2 in Appendix B, for 30 provinces, the annual average increase in opportunity cost associated with technical change is 4.8%. Instead, input change is associated with decreasing opportunity cost from 2003 to 2015, the size of annual decrease in cost also varies across the regions. The annual average decrease in opportunity cost associated with input change is 4.6%.

Comprehensively, technical change is the main contributor to the increase in opportunity cost, and input change decreases opportunity cost during the period. In fact, the increase in opportunity cost associated with technical change indicates that the unregulated frontier expands faster than regulated frontier. This finding implies that the unregulated technology or carbon-intensive technology has played a dominant role in China’s industrial sector in the past few years. Low-carbon or green production technology does not receive enough attention. The decline in opportunity cost attributed to input change shows that the industrial sectors has performed well in adjusting inputs towards the low-carbon mix during the period. That is, less carbon emissions per unit of industrial output are emitted compared with the carbon-intensive mix of inputs, which helps to decrease opportunity cost associated with regulations.

**Figure 6:**
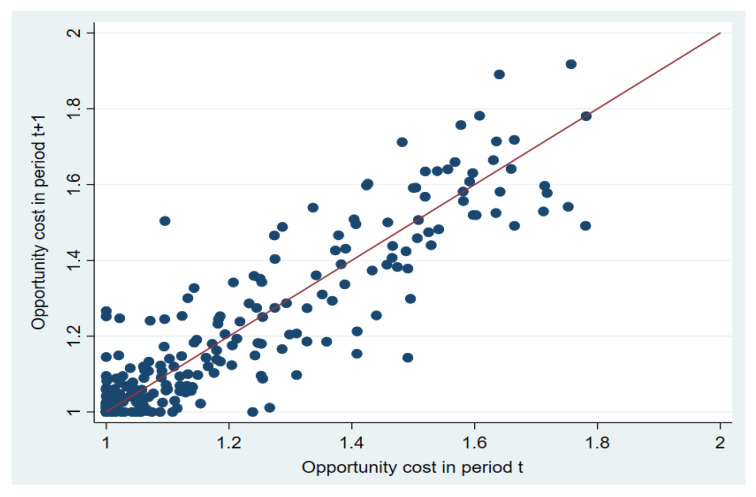
Change in opportunity cost by province over time. Note: The points in the figure represent the regional OC. The horizontal line represents the OC of the region in period *t*, and the vertical line represents the OC in period *t* + 1. The 45-degree line shows that regional OC does not change in two consecutive years. If the point is above the line, this means the OC increase from period *t* to *t* + 1; if the point is below the line, this means the OC decrease from *t* to *t* + 1.

Figure 6 illustrates the change in opportunity cost in any two consecutive years for 30 provinces. The 45-degree line shows that opportunity cost does not change in two consecutive years. A dot below (above) the 45-degree line suggests that the opportunity cost decreases (increases) relative to previous year. The results show that for the half points, opportunity cost declines compared to the previous year, respectively. For the other half points, we observe that opportunity cost increases compared to the previous years. For the entire period, as Table A2 shows, the geometric mean of opportunity cost change equals unity, which implies the opportunity cost almost remains the same between 2003 and 2015 from the national perspective. As we can observe from Table A2, this is because the negative effect of input change is canceled out by the positive impact of technical change on opportunity cost during the period.

### 4.2. Empirical Results from Mediation Analysis

#### 4.2.1. Baseline Results from Mediation Analysis

Table 3 reports the results of the mediation model. In Panel A, the first column shows a negative relationship between environmental regulations and inbound FDI in China’s industry. This is consistent with Millimet and Roy [42] who find significant negative effects of environmental stringency on inbound FDI in U.S. pollution-intensive industries. The negative coefficient in our results suggests that inbound FDI in China’s industrial sector may represent relatively dirty production technology, and the foreign multinationals shift their production which does not meet the environmental standards of their home country to China. This is in line with Cai et al. [43], who find that tougher environmental regulation leads to less inbound foreign direct investment in China.

As FDI represents less clean technology, industry needs to put more productive resources into the disposal of emissions when facing regulations, pushing up the opportunity cost. The positive coefficient of FDI on opportunity cost in column 3 confirms this. As shown in Panel B, environmental regulations have a significantly negative indirect effect on opportunity cost through FDI, estimated to be −0.107.

The second column in Panel A shows that environmental regulations spur R&D intensity in China’s industrial sector, which is similar to the results in Jaffe and Palmer [44] who find the positive relationship between environmental regulation and total R&D expenditures. Therefore, our results support the ‘weaker’ version of the Porter hypothesis that a properly designed environmental regulation may spur innovation.

The third column in Panel A further shows the insignificant negative relationship between R&D and opportunity cost. One may argue that environmental regulations force industry to allocate resources for the development of regulated production technology, which pushes the regulated frontier outward and shortens the distance between regulated frontier and unregulated frontier, resulting in a lower level of opportunity cost. However, our results suggest such a channel fails to be created, as shown in Panel B that the indirect effects of environmental regulations on opportunity cost through R&D is not significant.

Column 3 also shows the direct effect of environmental regulations on opportunity cost is significantly positive. Opportunity costs of abatement activities increase with stricter regulations, as environmental regulations force the industry to divert productive resources, which could be used for the production of goods, to dispose emissions. The total effect of environmental regulations on opportunity cost is positive, as in Panel B, implying opportunity cost can be higher with the stricter environmental regulations.

Our results show that the relationship between energy and opportunity costs is negative. This is consistent with the results from the previous section that input change decreases opportunity cost. The Chinese industrial sector performs well in adjusting inputs towards the low-carbon mix. The adjustment of energy input plays a crucial role in this process with the development of low-carbon technology, renewable energy, and the improvement of energy efficiency.

We also find a positive relationship between capital and opportunity cost. Given that industry has been a carbon-intensive sector in China in the past few decades, the tangible assets such as machinery invested in production activities are considered as less environment-friendly. One additional unit capital investment indicates extra carbon emission associated with increased industrial outputs. To reduce these carbon emissions under strict regulation, the loss in productive resource and thus industrial output will be higher, implying increasing opportunity cost.

Labor has little impact on the opportunity cost, and the main reason may lie in that the labor input does not influence the carbon emissions directly.

#### 4.2.2. Heterogeneous Effects by Region

We further examine the heterogeneous effects by region and Table 4 presents the corresponding results. Overall, we observe the significant regional heterogeneity. Specifically, the result of Panel A shows that environmental regulation has a significant crowding out effect on FDI in the western region, and no significant effect on FDI in other regions. From Panel B, we can see the significant positive effect of environmental regulation on R&D intensity in the eastern regions, but this effect is not observed in other regions. In Panel C, the significant positive effect of environmental regulation on opportunity cost is only found in the northern region of China. We also find the positive effect of capital on opportunity cost in the western and eastern regions. In addition, the significant negative effect of energy on opportunity cost is found in the eastern and southern regions. FDI has a significant positive effect on opportunity cost in the eastern region, whereas a significant negative effect is found in the southern region.

## 5. Conclusions

In this study, we first derive the opportunity cost associated with environmental regulations for the industrial sector in China and decompose the change of opportunity cost into two contributing factors via a non-parametric approach. The results show that opportunity cost attributed to environmental regulations is around zero for the industrial sector in Beijing, Tianjin, Hebei, Shanghai, Jiangsu, Shandong, Guangdong, and Hainan during the sample period. At the national level, there is no significant change on the opportunity cost in China’s industrial sector. The positive effect of technical change on opportunity cost was cancelled out by the negative effect of input change during the entire 2003–2015 period.

We then explore the extent to which environmental regulations affect opportunity cost through a parametric estimation. Specifically, we propose a mediation model to investigate the direct effect and indirect effect of environmental regulations on opportunity cost of carbon mitigation. We find that environmental regulations have a significantly positive direct effect (76.32%) and a significantly negative indirect effect (10.7%) on opportunity cost through foreign direct investment. Energy consumption has a negative impact on opportunity cost while capital can increase it.

These findings carry important policy implications for the low-carbon development of the industrial sector in China. Firstly, since opportunity cost can be higher with stricter environmental regulations, how to reduce opportunity cost arising from regulations is of importance for regional economy and firm profitability. We find that opportunity cost declines in Chinese industries are associated more with input change. This suggests that firms in the industrial sectors have adjusted their inputs accordingly to accommodate the increasing stringency of environmental regulation. Therefore, moving input mix towards low-carbon energy and capital when facing more stringent regulations could be an applicable way of alleviating the economic losses associated with regulations.

Moreover, the impacts of R&D in reducing the opportunity cost associated with environmental regulation are missing from our analyses. This may be due to the possible mis-allocation of resources in governmental planning. Therefore, future policy design shall consider measures that could reallocate resources towards the development of the regulated production technology, i.e., producing less carbon emissions per unit of industrial product. Such a regulatory-induced technical change can lead to a less carbon-intensive production process being capable of producing as many industrial products as the original free disposability (or less-regulated) technology. Thus, the opportunity cost of regulations (reduced industrial output due to abatement activities) can be reduced significantly.

Our analyses also show that during 2003–2015, inward FDI in China’s industrial sector may represent relatively dirty production technology. Therefore, regional governments must be careful in receiving “dirty” FDI which may increase local industrial pollution. Instead, the government should encourage the introduction of “clean” and high-technology FDI and strengthen the environmental monitoring of existing foreign-owned industrial enterprises. Domestic industrial firms should put more effort into absorbing these advanced and low-carbon technologies, and thus can help to decrease the total carbon emissions of the industrial sector. Finally, one limitation for this study is that we directly assume the separability condition holds and do not test the assumption, which should be further explored in future research.

## Figures and Tables

**Table 1:** Summary statistics (2003–2015).

Variable	N. Obs.	Unit	Mean	St. Dev.	Min	Max
y	390	10^8^ Yuan	16,835.735	22,188.596	247.9	130,177.787
CO_2_	390	10^4^ ton	15,188.005	10,957.742	985.885	54,612.751
Labor	390	10^4^ person	281.740	309.384	11.6	1568
Capital	390	10^8^ Yuan	4987.320	4433.920	197.3	26,791.931
Energy	390	10^4^ ton	5718.096	4152.231	365.143	20,765.304
ER	390	/	0.0022	0.0018	0.0004	0.0146
FDI	390	/	0.1729	0.1608	0.0126	0.6559
R&D	390	/	0.0089	0.0038	0.0018	0.0232

Note: Regional gross industrial product value *y*, foreign direct investment (FDI), labor and capital are derived from the China Statistical Yearbook and China Industrial Statistical Yearbook respectively. Energy consumption, environmental regulation (ER) variable and R&D expenditures are derived from the China Energy Statistical Yearbook, China Environmental Statistical Yearbook and China Statistical Yearbook on Science and Technology.

**Table 2:** Change in opportunity cost and its decomposition for provinces during 2003–2015.

Province	Δ*OC*	Δ*OC_TC_*	Δ*OC_IC_*	Province	Δ*OC*	Δ*OC_TC_*	Δ*OC_IC_*
Beijing	1.000	1.050	0.953	Henan	1.004	1.013	0.991
Tianjin	0.994	1.046	0.950	Hubei	0.977	1.053	0.928
Hebei	0.999	1.015	0.984	Hunan	0.981	1.012	0.970
Shanxi	1.023	1.078	0.949	Guangdong	1	1.0372	0.964
Inner Mongolia	0.974	1.038	0.938	Guangxi	0.985	1.033	0.954
Liaoning	1.001	1.025	0.976	Hainan	1	1.072	0.933
Jilin	1.001	1.043	0.960	Chongqing	0.999	1.007	0.992
Heilongjiang	1.023	1.076	0.951	Sichuan	0.985	1.036	0.951
Shanghai	1.003	1.037	0.967	Guizhou	1.003	1.061	0.946
Jiangsu	1	1.038	0.964	Yunnan	1.002	1.081	0.927
Zhejiang	1.005	1.028	0.978	Shaanxi	0.979	1.080	0.907
Anhui	0.997	1.022	0.976	Gansu	0.982	1.081	0.909
Fujian	0.994	1.021	0.974	Qinghai	1.022	1.120	0.912
Jiangxi	0.985	1.020	0.966	Ningxia	1.031	1.093	0.943
Shandong	1	1.030	0.971	Xinjiang	1.039	1.114	0.933

Note: Geometric mean of two-year pairs ΔOC, $\Delta OC_{TC}$ and $\Delta OC_{IC}$ from 2003–2004 to 2014–2015 represents the average annual change of opportunity cost, average annual change of opportunity cost associated with technical change and average annual change of opportunity cost associated with input change during 2003–2015. Percentage changes can be obtained by subtracting unity from values in the table.

**Table 3:** The results of the mediation model.

**Panel A**			
	(1) FDI	(2) R&D	(3) Opportunity cost
Environmental Regulation	−0.0622 ** (0.0320)	0.1059 *** (0.0396)	0.7632 ** (0.3453)
Energy	−0.2030 * (0.1062)	−0.1440 (0.0995)	−1.9899 *** (0.7492)
Capital	0.2417 ** (0.0993)	−0.2960 ** (0.1221)	3.6021 *** (1.1074)
Labor	−0.0283 (0.1062)	−0.4146 *** (0.1352)	−1.0377 (1.1534)
FDI			1.7252 *** (0.6583)
R&D			−0.2336 (0.5184)
Constant	−2.2018 *** (0.6598)	1.4084 * (0.7632)	−1.2786 (6.9350)
**Panel B**			
	Coefficient	Boot S.E.	95% Conf. Interval
*Indirect_FDI_*	−0.107	0.074	[−0.310,−0.010]
*Indirect_RD_*	−0.025	0.060	[−0.168, 0.077]
*Indirect_total_*	−0.132	0.087	[−0.338, 0.07]
Total effects	0.631	0.339	[0.015, 1.348]

Note: *Indirect_FDI_* represents the indirect effect of ER on OC through FDI. *Indirect_RD_* represents the indirect effect of ER on OC through R&D. *Indirect_total_* represents the total indirect effect of ER on OC. Total effects include direct effect and indirect effect. ***, ** and * denote significance at 10%, 5% and 1%; Bootstrap standard error in parentheses. Bias-corrected confidence intervals are reported in the table.

**Table 4:** Heterogeneous effects by region.

Panel A: FDI	West	East	North	Middle	South
Environmental regulation	−0.2279 *** (0.0666)	−0.0131 (0.1001)	0.0476 (0.0407)	0.0541 (0.1337)	−0.0239 (0.1003)
Energy	−0.1489 (0.2938)	−0.3091 (0.3555)	0.1538 (0.2241)	0.2412 (0.1910)	−0.1426 (0.1721)
Capital	0.4145 (0.3972)	0.0479 (0.3265)	0.0557 (0.1935)	0.5508 (0.4524)	0.1461 (0.2568)
Labor	0.4686 ** (0.2057)	0.1841 (0.3326)	−0.6889 ** (0.2769)	−2.6322 *** (0.6937)	−0.0575 (0.3739)
Constant	−7.8463 *** (2.0565)	0.1203 (2.2371)	−1.2851 (1.4503)	4.9908 * (2.7181)	−2.1861 (2.0888)
**Panel B: R&D**					
Environmental regulation	−0.0069 (0.0532)	0.2272 * (0.1305)	0.0786 (0.111)	−0.0517 (0.1987)	0.2301 (0.1968)
Energy	0.0410 (0.1536)	0.8381 *** (0.2934)	0.1006 (0.5451)	−0.2485 (0.2822)	−0.7124 (0.5740)
Capital	0.4812 ** (0.2441)	0.1921 (0.3257)	0.5460 (0.4211)	−1.0098 (0.6720)	0.3270 (0.6079)
Labor	−0.7135 *** (0.1910)	−1.8359 *** (0.3809)	−1.5403 ** (0.6413)	2.7142 *** (0.8988)	−0.6454 (0.6150)
Constant	−4.3580 (1.6303)	−1.4969 (2.0950)	−2.6035 (3.3341)	−7.9813 * (4.3548)	3.4627 (7.2453)
**Panel C: Opportunity Cost**					
Environmental regulation	0.5271 (0.6554)	1.3644 (1.0533)	0.1345 * (1.2936)	−0.8431 (1.2479)	0.0968 (1.1021)
Energy	−2.0442 (1.2771)	−7.4827 * (3.9471)	−3.7304 (6.2817)	−2.6985 (2.1587)	−6.3665 * (3.6657)
Capital	6.2315 *** (2.3997)	9.3207 *** (3.5381)	−5.7871 (4.6460)	4.3239 (3.5943)	3.7926 (2.7248)
Labor	−0.1565 (2.7068)	−4.3722 (5.1323)	4.5894 (10.8649)	9.2114 (8.7463)	−4.0951 (3.2376)
FDI	0.3966 (0.8501)	4.1294 * (2.4453)	3.6003 (7.0003)	4.8435 (3.1722)	−8.1509 ** (3.8888)
Constant	−19.4309 (21.4833)	22.1423 (22.4864)	91.2396 ** (39.6340)	−57.5722 * (33.3223)	25.3202 (35.2361)

Note: The West includes Chongqing, Sichuan, Guizhou, Yunnan, Shaanxi, Gansu, Qinghai, Ningxia and Xinjiang. The East includes Shanghai, Jiangsu, Zhejiang, Anhui, Fujian and Jiangxi. The Middle includes Beijing, Tianjin, Hebei, Shanxi, Shandong and Henan. The North includes Inner Mongolia, Liaoning, Jilin and Heilongjiang. The South includes Hubei, Hunan, Guangdong, Guangxi and Hainan. ***, ** and * denote significance at 10%, 5% and 1%; Bootstrap standard error in parentheses. Bias-corrected confidence intervals are reported in the table.

## Data Availability

Publicly available datasets were analyzed in this study, which could be obtained from the China Statistical Yearbook, China Industrial Statistical Yearbook, China Energy Statistical Yearbook, China Environmental Statistical Yearbook and China Statistical Yearbook on Science and Technology.

## Appendix A. Calculation

In this appendix, we employ directional distance function to construct the maximum desirable output *y*, and thus we have:

$$y_{u}^{t}\left(X^{t}\right)=y_{k^{\prime }}^{t}+{\vec{D}}_{u}^{t}\left(x_{k^{\prime }}^{t},y_{k^{\prime }}^{t},b_{k^{\prime }}^{t};g_{k^{\prime }}^{t}\right)y_{k^{\prime }}^{t};$$

$$y_{u}^{t+1}\left(X^{t}\right)=y_{k^{\prime }}^{t}+{\vec{D}}_{u}^{t+1}\left(x_{k^{\prime }}^{t},y_{k^{\prime }}^{t},b_{k^{\prime }}^{t};g_{k^{\prime }}^{t}\right)y_{k^{\prime }}^{t};$$

$$y_{u}^{t+1}\left(X^{t+1}\right)=y_{k^{\prime }}^{t+1}+{\vec{D}}_{u}^{t+1}\left(x_{k^{\prime }}^{t+1},y_{k^{\prime }}^{t+1},b_{k^{\prime }}^{t+1};g_{k^{\prime }}^{t+1}\right)y_{k^{\prime }}^{t+1};$$

$$y_{u}^{t}\left(X^{t+1}\right)=y_{k^{\prime }}^{t+1}+{\vec{D}}_{u}^{t}\left(x_{k^{\prime }}^{t+1},y_{k^{\prime }}^{t+1},b_{k^{\prime }}^{t+1};g_{k^{\prime }}^{t+1}\right)y_{k^{\prime }}^{t+1};$$

$$y_{r}^{t}\left(X^{t}\right)=y_{k^{\prime }}^{t}+{\vec{D}}_{r}^{t}\left(x_{k^{\prime }}^{t},y_{k^{\prime }}^{t},b_{k^{\prime }}^{t};g_{k^{\prime }}^{t}\right)y_{k^{\prime }}^{t};$$

$$y_{r}^{t+1}\left(X^{t}\right)=y_{k^{\prime }}^{t}+{\vec{D}}_{r}^{t+1}\left(x_{k^{\prime }}^{t},y_{k^{\prime }}^{t},b_{k^{\prime }}^{t};g_{k^{\prime }}^{t}\right)y_{k^{\prime }}^{t};$$

$$y_{r}^{t+1}\left(X^{t+1}\right)=y_{k^{\prime }}^{t+1}+{\vec{D}}_{r}^{t+1}\left(x_{k^{\prime }}^{t+1},y_{k^{\prime }}^{t+1},b_{k^{\prime }}^{t+1};g_{k^{\prime }}^{t+1}\right)y_{k^{\prime }}^{t+1};$$

(A1) $$y_{r}^{t}\left(X^{t+1}\right)=y_{k^{\prime }}^{t+1}+{\vec{D}}_{r}^{t}\left(x_{k^{\prime }}^{t+1},y_{k^{\prime }}^{t+1},b_{k^{\prime }}^{t+1};g_{k^{\prime }}^{t+1}\right)y_{k^{\prime }}^{t+1}.$$

$y_{k^{\prime }}^{t}$ and $y_{k^{\prime }}^{t+1}$ are actual good output (i.e., gross industrial product value) of industrial sector in region *k*′ in period *t* and *t* + 1 respectively. ${\vec{D}}_{u}^{t}\left(x_{k^{\prime }}^{t},y_{k^{\prime }}^{t},b_{k^{\prime }}^{t};g_{k^{\prime }}^{t}\right)$ and ${\vec{D}}_{u}^{t+1}\left(x_{k^{\prime }}^{t+1},y_{k^{\prime }}^{t+1},b_{k^{\prime }}^{t+1};g_{k^{\prime }}^{t+1}\right)$ represent the unregulated directional distance functions in period *t* and *t* + 1 respectively, and ${\vec{D}}_{u}^{t+1}\left(x_{k^{\prime }}^{t},y_{k^{\prime }}^{t},b_{k^{\prime }}^{t};g_{k^{\prime }}^{t}\right)$ and ${\vec{D}}_{u}^{t}\left(x_{k^{\prime }}^{t+1},y_{k^{\prime }}^{t+1},b_{k^{\prime }}^{t+1};g_{k^{\prime }}^{t+1}\right)$ are mix-period unregulated directional distance functions. Similarly, the corresponding regulated directional distance functions can be explained in the same way.

The unregulated directional distance function ${\vec{D}}_{u}^{t}\left(x_{k^{\prime }}^{t},y_{k^{\prime }}^{t},b_{k^{\prime }}^{t};g_{k^{\prime }}^{t}\right)$ can be calculated as follows:

$${\vec{D}}_{u}^{t}\left(x_{k^{\prime }}^{t},y_{k^{\prime }}^{t},b_{k^{\prime }}^{t};g_{k^{\prime }}^{t}\right)=\mathrm{Max}\;\beta$$

$$\;s.t.\;\sum_{k=1}^{K}z_{k}y_{k}^{t}\geq y_{k^{\prime }}^{t}+\beta y_{k^{\prime }}^{t};$$

$$\;\sum_{k=1}^{K}z_{k}b_{k}^{t}\geq b_{k^{\prime }}^{t}-\beta b_{k^{\prime }}^{t};$$

$$\;\sum_{k=1}^{K}z_{k}x_{kn}^{t}\leq x_{k^{\prime }n}^{t};\;n=1,\;2,\;3$$

(A2) $$\;\sum_{k=1}^{K}z^{k}=1,\;z_{k}\geq 0,\;k=1,\ldots ,K$$

The calculation of ${\vec{D}}_{u}^{t+1}\left(x_{k^{\prime }}^{t+1},y_{k^{\prime }}^{t+1},b_{k^{\prime }}^{t+1};g_{k^{\prime }}^{t+1}\right)$ is the same as in Equation (A2) except that the superscript *t* is substituted by *t* + 1.

The mix-period directional distance function can be estimated as:

$${\vec{D}}_{u}^{t+1}\left(x_{k^{\prime }}^{t},y_{k^{\prime }}^{t},b_{k^{\prime }}^{t};g_{k^{\prime }}^{t}\right)=\mathrm{Max}\;\beta$$

$$\;s.t.\;\sum_{k=1}^{K}z_{k}y_{k}^{t+1}\geq y_{k^{\prime }}^{t}+\beta y_{k^{\prime }}^{t};$$

$$\;\sum_{k=1}^{K}z_{k}b_{k}^{t+1}\geq b_{k^{\prime }}^{t}-\beta b_{k^{\prime }}^{t};$$

$$\;\sum_{k=1}^{K}z_{k}x_{kn}^{t+1}\leq x_{k^{\prime }n}^{t};\;n=1,\;2,\;3$$

(A3) $$\;\sum_{k=1}^{K}z^{k}=1,\;z^{k}\geq 0,\;k=1,\ldots ,K$$

and,

$${\vec{D}}_{u}^{t}\left(x_{k^{\prime }}^{t+1},y_{k^{\prime }}^{t+1},b_{k^{\prime }}^{t+1};g_{k^{\prime }}^{t+1}\right)=\mathrm{Max}\;\beta$$

$$\;s.t.\;\sum_{k=1}^{K}z_{k}y_{k}^{t}\geq y_{k^{\prime }}^{t+1}+\beta y_{k^{\prime }}^{t+1};$$

$$\;\sum_{k=1}^{K}z_{k}b_{k}^{t}\geq b_{k^{\prime }}^{t+1}-\beta b_{k^{\prime }}^{t+1};$$

$$\;\sum_{k=1}^{K}z_{k}x_{kn}^{t}\leq x_{k^{\prime }n}^{t+1};\;n=1,\;2,\;3$$

(A4) $$\;\sum_{k=1}^{K}z^{k}=1,\;z^{k}\geq 0,\;k=1,\ldots ,K$$

Replacing the inequality constraints of the bad outputs with equality ones in Equations (A2)–(A4), we can obtain the corresponding regulated directional distance functions.

## Appendix B. Additional Empirical Results

In this appendix, we report part of the empirical results in Section 4.

**Table A1:** Summary statistics of estimated opportunity cost from 2003 to 2015 (full sample).

Region	Mean	St. Dev.	Min	Max	Region	Mean	St. Dev.	Min	Max
Beijing	1.001	0.003	1	1.009	Henan	1.058	0.069	1	1.191
Tianjin	1.006	0.021	1	1.074	Hubei	1.128	0.113	1	1.409
Hebei	1.001	0.004	1	1.014	Hunan	1.026	0.073	1	1.255
Shanxi	1.575	0.130	1.373	1.891	Guangdong	1	0	1	1
Inner Mongolia	1.050	0.116	1	1.408	Guangxi	1.158	0.098	1.038	1.359
Liaoning	1.031	0.053	1	1.149	Hainan	1	0	1	1
Jilin	1.044	0.055	1	1.145	Chongqing	1.017	0.032	1	1.092
Heilongjiang	1.610	0.157	1.337	1.918	Sichuan	1.092	0.089	1	1.327
Shanghai	1.004	0.011	1	1.042	Guizhou	1.447	0.106	1.287	1.635
Jiangsu	1	0	1	1	Yunnan	1.148	0.071	1	1.245
Zhejiang	1.037	0.021	1.001	1.069	Shaanxi	1.426	0.172	1.207	1.752
Anhui	1.047	0.038	1	1.120	Gansu	1.345	0.155	1.124	1.665
Fujian	1.014	0.034	1	1.108	Qinghai	1.136	0.111	1	1.300
Jiangxi	1.080	0.098	1	1.310	Ningxia	1.414	0.263	1	1.782
Shandong	1	0	1		Xinjiang	1.300	0.212	1	1.591

**Table A2:** Δ*OC*, Δ*OC_TC_* and Δ*OC_IC_* for two-year pairs (30 provinces).

Two-Year Pairs	Δ*OC*	Δ*OC_TC_*	Δ*OC_IC_*	Two-Year Pairs	Δ*OC*	Δ*OC_TC_*	Δ*OC_IC_*
2003–2004	0.949	1.075	0.882	2009–2010	1.012	1.056	0.958
2004–2005	0.968	1.033	0.937	2010–2011	0.993	1.088	0.912
2005–2006	1.026	1.061	0.967	2011–2012	0.992	1.023	0.970
2006–2007	1.016	1.082	0.939	2012–2013	0.985	1.015	0.971
2007–2008	1.021	1.046	0.976	2013–2014	1.018	1.036	0.983
2008–2009	0.978	1.006	0.973	2014–2015	1.040	1.061	0.980
				Geometric mean	1.000	1.048	0.954

Note: Geometric means of 30 provinces for two-year pairs are reported in the table; percentage changes can be obtained by subtracting unity from values in the table.
